# Tumor Cell Plasticity in Equine Papillomavirus-Positive Versus-Negative Squamous Cell Carcinoma of the Head and Neck

**DOI:** 10.3390/pathogens11020266

**Published:** 2022-02-18

**Authors:** Carina Strohmayer, Andrea Klang, Stefan Kummer, Ingrid Walter, Christoph Jindra, Christiane Weissenbacher-Lang, Torben Redmer, Sibylle Kneissl, Sabine Brandt

**Affiliations:** 1 Clinical Unit of Diagnostic Imaging, Department for Companion Animals and Horses, University of Veterinary Medicine, 1210 Vienna, Austria; carina.strohmayer@vetmeduni.ac.at (C.S.); sibylle.kneissl@vetmeduni.ac.at (S.K.); 2 Institute of Pathology, Department of Pathobiology, University of Veterinary Medicine, 1210 Vienna, Austria; andrea.klang@vetmeduni.ac.at (A.K.); christiane.weissenbacher-lang@vetmeduni.ac.at (C.W.-L.); 3 VetCore Facility for Research, University of Veterinary Medicine, 1210 Vienna, Austria; stefan.kummer@vetmeduni.ac.at (S.K.); ingrid.walter@vetmeduni.ac.at (I.W.); 4 Institute of Morphology, Department of Pathobiology, University of Veterinary Medicine, 1210 Vienna, Austria; 5 Research Group Oncology (RGO), Clinical Unit of Equine Surgery, Department for Companion Animals and Horses, University of Veterinary Medicine, 1210 Vienna, Austria; christoph.jindra@vetmeduni.ac.at; 6 Institute of Medical Biochemistry, Department of Biomedical Sciences, University of Veterinary Medicine, 1210 Vienna, Austria; torben.redmer@vetmeduni.ac.at

**Keywords:** horse, HNSCC, papillomavirus, immunohistochemistry, immunofluorescence, tumor cell plasticity

## Abstract

Squamous cell carcinoma of the head and neck (HNSCC) is a common malignant tumor in humans and animals. In humans, papillomavirus (PV)-induced HNSCCs have a better prognosis than papillomavirus-unrelated HNSCCs. The ability of tumor cells to switch from epithelial to mesenchymal, endothelial, or therapy-resistant stem-cell-like phenotypes promotes disease progression and metastasis. In equine HNSCC, PV-association and tumor cell phenotype switching are poorly understood. We screened 49 equine HNSCCs for equine PV (EcPV) type 2, 3 and 5 infection. Subsequently, PV-positive versus -negative lesions were analyzed for expression of selected epithelial (keratins, β-catenin), mesenchymal (vimentin), endothelial (COX-2), and stem-cell markers (CD271, CD44) by immunohistochemistry (IHC) and immunofluorescence (IF; keratins/vimentin, CD44/CD271 double-staining) to address tumor cell plasticity in relation to PV infection. Only EcPV2 PCR scored positive for 11/49 equine HNSCCs. IHC and IF from 11 EcPV2-positive and 11 EcPV2-negative tumors revealed epithelial-to-mesenchymal transition events, with vimentin-positive cells ranging between <10 and >50%. CD44- and CD271-staining disclosed the intralesional presence of infiltrative tumor cell fronts and double-positive tumor cell subsets independently of the PV infection status. Our findings are indicative of (partial) epithelial–mesenchymal transition events giving rise to hybrid epithelial/mesenchymal and stem-cell-like tumor cell phenotypes in equine HNSCCs and suggest CD44 and CD271 as potential malignancy markers that merit to be further explored in the horse.

## 1. Introduction

Squamous cell carcinoma (SCC) is a common epithelial tumor type in humans and animals that arises from cutaneous or mucosal keratinocytes. In humans, virtually 100% of cervical carcinomas, about 50% of anogenital SCCs, and approximately 25% of head and neck SCCs (HNSCC) are causally associated with infection by carcinogenic papillomaviruses (PVs) [1]. These PVs termed high-risk human PVs (hrHPVs) belong to the genus α-PVs. Despite their high genetic heterogeneity, hrHPVs share common features. These include their ability to transform normal keratinocytes into highly proliferative neoplastic cells by a concerted action of the oncoproteins E6 and E7 [2], and to escape from immune surveillance via E5 oncoprotein-mediated downregulation of the MHC class I [3].

HNSCCs refer to SCCs of the nasal and oral cavity, the larynx, and the pharynx, i.e., the naso-, oro-, and hypopharynx. It is widely accepted today that hrHPV-induced and -unrelated HNSCCs constitute two different disease entities. Whilst hrHPV-associated HNSCCs predominantly affect the oropharynx, including the tonsils and base of the tongue, hrHPV-unrelated tumors have no defined predilection sites in the HN region and develop upon exposure to various carcinogens, e.g., alcohol and tobacco [4,5]. In addition, there is evidence of Epstein-Barr virus—a γ-Herpesvirus—contributing to the onset and progression of certain HNSCC subtypes, such as oral SCC [6].

SCCs are malignant tumors, i.e., they have the potential to invade surrounding tissues and establish metastases in other parts of the body. Yet, hrHPV-positive and -negative HNSCCs have different clinical behavior in terms of infiltrative growth and metastasis, with hrHPV-induced lesions showing higher sensitivity to multiple-type therapy and thus having a better prognosis than their hrHPV-unrelated counterparts [7].

In horses and other equids, SCCs preferentially develop at muco-cutaneous junctions, notably the anogenital, ocular, and HN region [8]. Meanwhile, there is ample evidence that equine papillomavirus type 2 (EcPV2) infection causes virtually 100% of anogenital SCCs [9,10]. In contrast, ocular SCCs seem to be unrelated to PV infection. There is general agreement that overexposure to UV-radiation constitutes a major risk factor for ocular SCC development [8,11]. The etiology of equine HNSCC is still poorly understood. Occasional detection of EcPV2 DNA from HNSCCs reported by different groups suggests that a subset of these tumors may be associated with EcPV2 infection [12,13]. However, in-depth research is needed to ascertain that EcPV2 has an active role in the development and progression of some HNSCCs.

Cancer progression and metastasis is a multistep event initiated by genetic and nongenetic cell transforming processes. The latter mechanism, termed cellular plasticity enables the rapid switching of tumor cell phenotypes in response to extracellular cues such as changes in the composition of growth factor or oxygen supply [14]. Consequently, cellular transformation modulates cell–cell interactions, enabling tumor cells to detach from the primary mass, invade and migrate through the extracellular matrix (ECM), enter the microvasculature, and spread via the blood stream and the lymphatics [15,16]. This cascade of events closely resembles an embryologic cellular program termed epithelial–mesenchymal transition (EMT). This program is activated in embryonic development and wound healing and orchestrates the conversion of various types of epithelial cells into mesenchymal cells [15,16,17,18]. EMT can be grossly characterized by the loss of typical epithelial properties (e.g., apical–basolateral polarization, basement membrane integrity, cell– cell adhesion) and the gain of mesenchymal characteristics. This transition is mediated by E-cadherin suppression, allowing EMT transcription factors to promote enhanced expression of mesenchymal proteins. The latter, including, e.g., N-cadherin, vimentin, and matrix metalloproteinases (MMPs), promote ECM degradation, cell motility, invasion, and metastasis [16,19]. EMT-mediated changes confer poor immunogenicity, prevent senescence and apoptosis, and enhance the migratory capacity and invasiveness of tumor cells, thus promoting infiltrative growth and metastasis of solid tumors, including HNSCCs [20,21,22,23]. Under in vitro conditions, human tumor cells commonly undergo complete EMT as represented by the E- to N-cadherin switch. Under natural in vivo conditions however, human epithelial cancer cells preferentially switch to a hybrid E/M phenotype in a process termed partial EMT (pEMT), which is regulated by ECM components, soluble factors, and exosomes [24]. In human HNSCC, pEMT is well-documented [25]. It is characterized by the concurrent expression of both epithelial and mesenchymal proteins that allows epithelial/mesenchymal (E/M) hybrid tumor cells to migrate collectively as cell clusters. E/M-phenotype tumor cells are thus regarded as best-suited for metastasis [24,25].

In the past years, it has been shown that EMT not only allows tumor cells to acquire a hybrid E/M or mesenchymal phenotype, but is also associated with a second program promoting transition of epithelial tumor cells to a “cancer stem(-like) cell” (CSC) phenotype [21,26]. CSCs are long-lived tumor cells with typical stem-cell properties that notably include the abilities for self-renewal and multidirectional differentiation [27]. CSCs show pronounced resistance to stress factors such as DNA damage [28], reactive oxygen species [29], or hypoxia [30], and by this, to various therapeutic approaches including chemo- and radiation therapy. Thus, CSCs are recognized as crucial promoters of tumor progression and reoccurrence, immune evasion, and metastasis [31,32]. This in turn explains why CSCs are also termed tumor-initiating cells (TICs) [33].

Meanwhile, an increasing number of reports help pave the way towards a better understanding of human CSC biology in cancer diseases, including HNSCC [31,34,35,36,37].

In horses, the etiology of HNSCC is largely unclear, and there is only scarce information on cancer cell plasticity in this tumor type. Only three studies have addressed EMT in equine SCC so far. Suárez-Bonnet and colleagues provided consistent evidence of EMT events occurring at the infiltration front of equine penile SCCs [38]. This finding was further corroborated by Armando et al. [39], who also reported on EMT in the case of an EcPV2-positive laryngeal SCC [40]. No information is available on CSCs in equine HNSCCs or any other equine tumor disease so far.

The higher multidrug-sensitivity of hrHPV-induced HNSCCs in comparison to their hrHPV-unrelated counterparts, and the crucial role of EMT and related CSCs as promotors of malignant progression and multidrug resistance (MDR) are suggestive of PV oncoproteins mitigating EMT processes. Given the still-prevailing paucity of knowledge on the pathobiological events underlying equine HNSCC development and metastatic dissemination, the role of EMT and related CSC in this context, and the impact of PV infection on EMT, the objective of this study was (i) to screen a series of equine HNSCCs for the presence of EcPV DNA, and (ii) comparatively analyze EcPV-positive versus -negative lesions for expression of selected epithelial (keratins, β-catenin), mesenchymal (vimentin), endothelial (COX-2), and stem-cell markers (CD271, CD44) by immunohistochemical (IHC) and immunofluorescent staining (IF). The major findings of this study indicate that equine HNSCCs can be categorized into EcPV2-related and -unrelated HNSCC subtypes, at a similar ratio as determined for hrHPV-induced versus -unrelated HNSCCs in humans. We provide evidence of (p)EMT occurring in virtually all lesions to a various extent, and, importantly, of the presence CD44^+^ CD271^+^ tumor cell subsets that likely represent CSCs. No significant correlation between (p)EMT/CSC- and EcPV2 infection status was observed.

## 2. Results

### 2.1. Twenty-Two Percent of HNSCC Samples Harbor EcPV2 DNA

**Figure 1:**
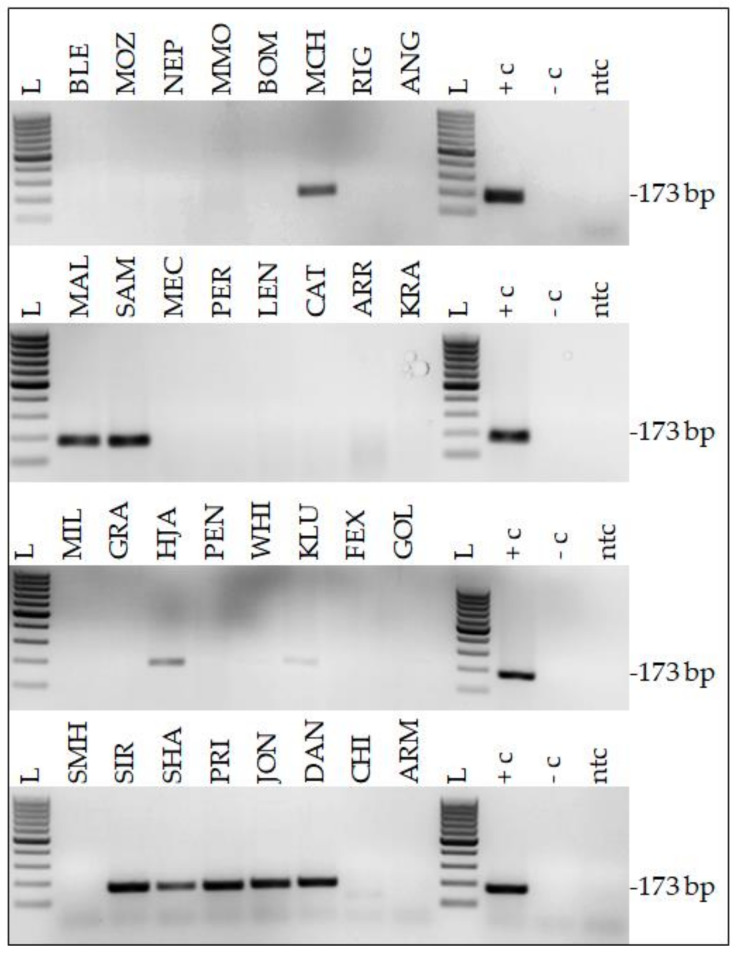
A subset of equine HNSCCs harbored EcPV2 DNA. Amplification products (16 µL) were run on 2% TAE gels and visualized by ethidium bromide staining. PCR results are exemplarily shown for 32/49 samples, with PCR yielding amplicons of anticipated size (173 bp) in 10 of the 32 presented cases. L: Gene Ruler 100 bp DNA ladder (Thermo Scientific); +c: positive control (EcPV2-positive equine penile SCC); -c: negative control (PV-free equine skin DNA); ntc: no template control (sterile water).

DNA isolated from 49 equine HNSCCs (49 equine patients) was assessed for the presence of EcPV type 2, 3, and 5 DNA on the grounds that these three EcPV2 types are reported to occur in equine SCC [9,10,41,42]. As expected, EcPV2 PCR from 4/15 native lesions previously shown to contain EcPV2 DNA [13] yielded amplicons of anticipated size (173 bp; Table 1; Figure 1). DNA extracted from another native tumor (VLU) also scored positive for this virus type. From the DNA aliquots derived from 27 FFPE tumor samples, six tested positive by EcPV2 PCR (Table 1; Figure 1). None of the tumor DNA samples harbored EcPV3 or EcPV5 DNA (not shown). PCR from positive, negative, and no-template controls included in every reaction yielded anticipated results, thus confirming the experimental accuracy and the validity of results (Figure 1).

### 2.2. Histopathological Findings

Sections from six noncornifying and 16 cornifying HNSCCs were subjected to histopathological examination. The well-differentiated cornifying tumors consisted of broad and anastomosing trabeculae, cords, and islands of tumor cells displaying varying degrees of keratinization including concentrically laminated keratin formation—so-called keratin pearls. Tumor cells were characterized by minimal cellular atypia, mild anisocytosis and anisokaryosis, and a moderate mitotic index. The six poorly differentiated, noncornifying HNSCCs exhibited irregular trabeculae, nests, and clusters of tumor cells without keratinization that showed moderate to marked cellular atypia and pronounced anisocytosis. Interspersed dyskeratotic cells were also noted. The tumor cells displayed prominent nuclear pleomorphism with anisokaryosis, high mitotic index including pathologic mitotic figures, and one or multiple prominent nucleoli. Multinucleated tumor cells and macronuclei were occasionally observed. Tumorous infiltrates were commonly accompanied by multifocal areas of necrosis, proliferation of fibrous tissue, desmoplasia and infiltration of the adjacent tumor stroma by lymphocytes, plasma cells and, in some cases, numerous neutrophils.

Individual tumor locations, evidence and location of metastasis, and additional findings such as osteolysis of adjacent structures are provided in Table 1.

### 2.3. Immunohistochemical Staining Reveals Tumor Cell Plasticity in Equine HNSCC

#### 2.3.1. Keratin (KRT)

**Figure 2:**
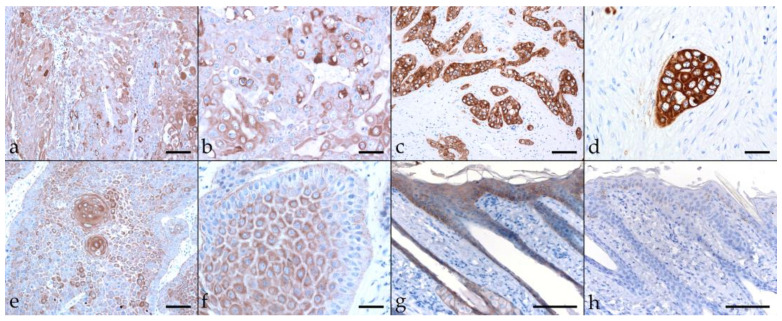
Cytokeratin labeling of EcPV2-positive and EcPV2-negative HNSCC sections. The figure depicts a representative selection of immunohistochemical staining results. a/b: Detection of KRTs in an EcPV2-positive, gingivopalatinal HNSCC (JON). Note the patchy distribution and varying intensity of KRT labeling, and the pronounced disorganization within the lesion. (**a**) Bar = 80 µm, (**b**) bar = 40 µm. c/d: diffuse, intensive KRT labeling of cell islets in an EcPV2-negative, palatinal squamous cell carcinoma (LUK). (**c**) Bar = 80 µm, (**d**) bar = 40 µm. (**e**,**f**): Mild to moderate KRT labeling in an EcPV2-positive, mandibular HNSCC (SIR) revealing the presence of keratin pearls (**e**), a typical feature of well differentiated SCCs, and signal intensity increasing from the basal to the squamous layers (**f**); (**e**) bar = 80 µm, (**f**) bar = 40 µm. (**g**): KRT labeling (positive control), and (**h)**: mock labeling of equine skin (no-primary Ab control). Bars = 100 µm.

Keratins (KRT) are a group of intermediate filament cytoskeletal proteins. Stratified epithelial KRTs can be classified as Type 1 (or low molecular weight; LMW) acidic, and type 2 (or high MW; HMW) basic KRTs [43,44]. EMT-driven changes in cellular morphology are based on cytoskeletal intermediate filament rearrangements mediated by modulated KRT expression [45]. Consequently, we first assessed EcPV2-positive and -negative equine HNSCCs for KRT expression using a pan-KRT antibody cocktail (AE1, AE3) confirmedly reacting with basic KRTs 1, 3, 4, 5, 6, and 8, and acidic KRTs 10, 14, 15, 16, and 19 according to the manufacturer (Cell Marque). All lesions scored positively to various extents, with KRT expression being confined to the cytoplasm, the physiological location of KRT intermediate filaments [44]. Various KRT staining intensities and patterns were noted, as exemplarily depicted in Figure 2. A pronouncedly central signal was observed in case of sections from four EcPV2-positive, and five EcPV2-negative HNSCCs (Table 2). Equine skin sections used as positive control exhibited intense KRT-labeling of mucosal epithelial cells. No signal was exhibited by the no-primary Ab control section (Figure 2).

#### 2.3.2. β-Catenin

**Figure 3:**
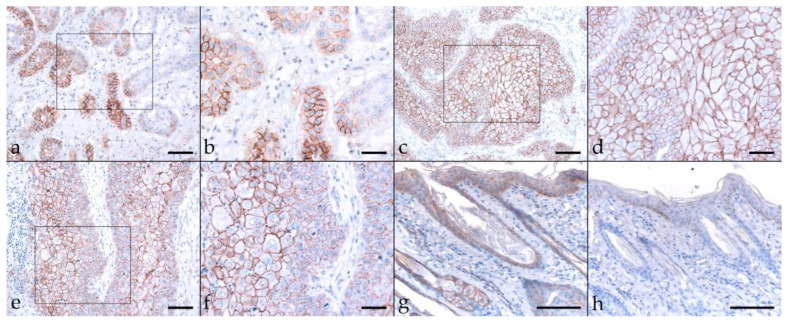
β-catenin labeling of EcPV2-positive and EcPV2-negative HNSCC sections. The figure depicts a representative selection of β-catenin labeling results. (**a**,**b**): patchy, strong membranous β-catenin labeling of >50% of tumor cells in an EcPV2-negative oral SCC (KRA). (**a**) Bar = 150 µm, (**b**) bar = 80 µm. (**c**,**d**): Strong, diffuse β-catenin labeling in an EcPV2-positive, pharyngeal SCC (SAM). (**c**) Bar = 150 µm, (**d**) bar = 80 µm. (**e**,**f**): Centrally focused β-catenin labeling pattern in an EcPV-positive, maxillary sinusoidal SCC (HJA). (**e**) Bar = 150 µm, (**f**) bar = 80 µm. (**g**,**h**): Positive and negative control, i.e., β-catenin labeled (**g**) and mock labeled ((**h**); no primary Ab) equine skin sections. Bars = 100 µm. Framed areas are presented at higher magnification.

Epithelial cell adhesion is mediated by E-cadherin. It builds on the intracellular attachment to the actin cytoskeleton by interaction with catenins, including β-catenin. There is growing evidence that reduced assembly of membranous β-catenin in favor of nuclear expression of this protein is associated with human head-and-neck and equine SCC invasiveness and metastasis [39,40,46,47,48]. Hence, we also stained equine HNSCCs for β-catenin, revealing low-to-pronounced membranous expression in most tumor sections, with diffuse (16 tumors) predominating over patchy signal distribution (two EcPV2-positive and EcPV2-negative HNSCCs) (Table 2). In some cases, β-catenin labeling was particularly intense within infiltration fronts of lesions. Cytoplasmic/nuclear β-catenin expression was only observed occasionally. Equine skin sections used as positive control exhibited β-catenin labeling whilst no-primary Ab control sections of equine skin scored negatively (Figure 3).

#### 2.3.3. Vimentin

**Figure 4:**
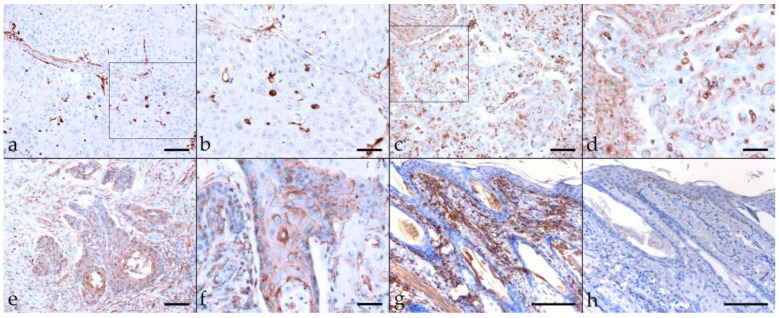
Vimentin staining of EcPV2-positive and EcPV2-negative HNSCC sections. The figure depicts a representative selection of vimentin labeling results. (**a**,**b**): Detection of vimentin in an EcPV2-positive maxillary sinusoidal SCC (HJA) in <10% of tumor cells, (**a**) bar = 80 µm, (**b**) bar = 40 µm. (**c**,**d**): Vimentin staining of <50% of tumor cells in an EcPV2-negative sinusoidal SCC (MMO), (**c**) bar = 80 µm, (**d**) bar = 40 µm. (**e**,**f**): Detection of vimentin in >50% of tumor cells in an EcPV2-negative maxillary sinusoidal SCC (FIL), (**e**) bar = 80 µm, (**f**) bar = 40 µm. (**g**,**h**): Vimentin (**g**) and mock labeling (**h**) of mesenchymal equine skin cells, bar = 100 µm. Framed areas are presented at higher magnification.

EMT is inter alia characterized by loss of cell adhesion properties and acquisition of mesenchymal features via downregulation of E-cadherin. This allows EMT transcription factors to promote expression of mesenchymal proteins including vimentin that confer the ability on tumor cells to cross tissue barriers. Therefore, vimentin is considered as a reliable EMT marker [16,19,49]. Vimentin labeling yielded an intensive cytoplasmic signal in most cases. The signal exhibited a patchy distribution. Percentages of vimentin-positive cells greatly varied, ranging from <10% (eight EcPV2^+^ and five EcPV2^−^ tumors), and <50% (two EcPV2^+^ and five EcPV2^−^ tumors), to >50% (EcPV2^+^ tumor KLU, and EcPV2^−^ tumor FIL) (Table 2). The equine skin positive control exhibited vimentin labeling of mesenchymal cells. No-primary Ab equine skin sections scored consistently negative (Figure 4).

#### 2.3.4. Cyclooxygenase-2 (COX-2)

**Figure 5:**
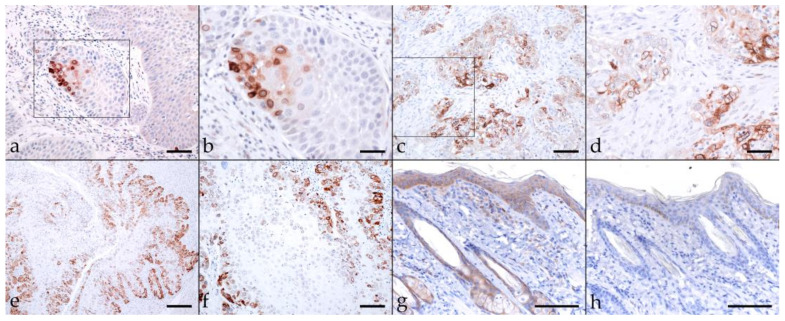
COX-2-staining of EcPV2-positive and EcPV2-negative HNSCC sections. The figure depicts a representative selection of COX-2 staining results. (**a**,**b**): Strong membranous and cytoplasmic COX-2 staining of <10% of tumor cells in an EcPV2-positive, mandibular SCC (SIR), (**a**) bar = 80 µm, (**b**) bar = 40 µm. (**c**,**d**): COX-2 staining of most tumor cells in an EcPV2-negative, sinusoidal SCC (MMO), (**c**) bar = 80 µm, (**d**) bar = 40 µm. (**e**,**f**): Pronounced COX-2-staining of cells within the infiltration front in an EcPV2-negative, maxillary sinusoidal SCC (PER), (**e**) bar = 150 µm, (**f**) bar = 80 µm. (**g**,**h**): Positive (**g**) and negative control (**h**) (equine skin ± primary Ab), bar = 100 µm. Framed areas are presented at higher magnification.

The enzyme COX-2 is known to promote the development and progression of various cancers including HNSCCs via pleiotropic functions that also include the induction of EMT [50]. COX-2-staining of equine HNSCC sections revealed a cytoplasmic and/or membranous localization of the enzyme, with variable staining intensities observed in <10% to >50% of tumor cells. In two EcPV2-positive (VLU, KLU) and two EcPV2-negative HNSCCs (PER, VAL), COX-2-staining was most pronounced within tumorous infiltration fronts (Table 3). Equine skin sections that served as positive controls exhibited labeling of COX-2 antigen, whereas no signal was exhibited when omitting the primary Ab (Figure 5).

#### 2.3.5. CD44

**Figure 6:**
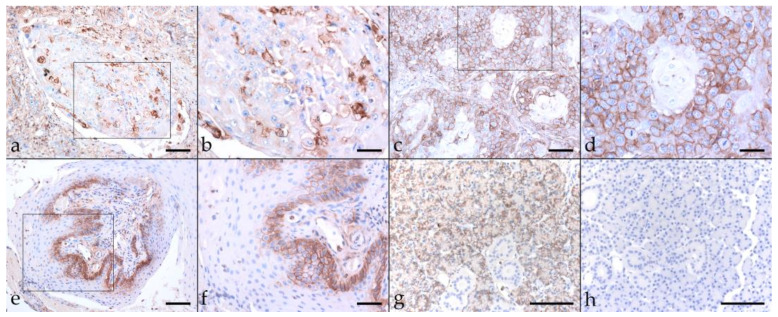
CD44-staining of EcPV2-positive and EcPV2-negative HNSCC sections. The figure depicts a representative selection of CD44-staining results. (**a**,**b**): patchy, cytoplasmic, and membranous CD44-staining in an EcPV2-negative, lingual SCC (BOM), (**a**) bar = 150 µm, (**b**) bar = 80 µm. (**c**,**d**): Patchy, membranous CD44-staining of >50% of tumor cells in an EcPV2-negative oral SCC (MEC), (**c**) bar = 150 µm, (**d**) bar = 80 µm. (**e**,**f**): Strong marginal CD44-staining pattern in an EcPV2-postive, pharyngeal SCC (SAM), (**e**) bar = 150 µm, (**f**) bar = 80 µm. (**g**,**h**): CD44- (**g**, positive control) and mock-staining (**h**, negative control) of an equine salivary gland section, bar = 100 µm. Framed areas are presented at higher magnification.

CD44 is a transmembrane glycoprotein, which acts as a major hyaluronan (HA) receptor, and by this, mediates cell–cell and cell–ECM interactions. In many tumors, CD44 has been recognized as a CSC marker [51]. CD44-staining of EcPV2-positive and -negative equine HNSCCs yielded membranous signals of varying intensity, and a predominantly patchy distribution, with <50% to >50% of tumor cells scoring positive. Of note, 12 sections displayed an aberrant, cytoplasmic signal irrespective of the EcPV2 infection status. In some cases, staining was evident in the margins of tumor islets (Table 3). Basal equine salivary gland cells (positive control) scored CD44-positive, no-primary Ab sections tested negative (Figure 6).

#### 2.3.6. CD271 (p75^NTR^)

**Figure 7:**
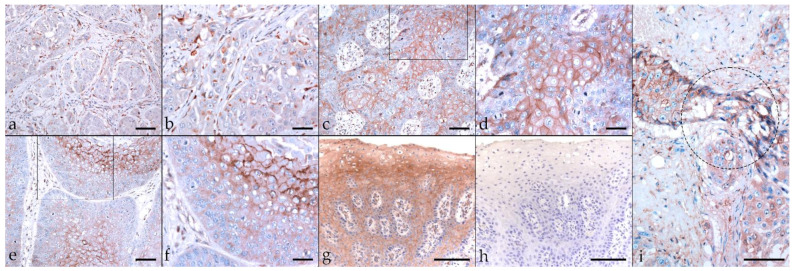
CD271 labeling of EcPV2-positive and EcPV2-negative HNSCC sections. The figure depicts a representative selection of CD271 labeling results. (**a**,**b**): Cytoplasmic CD271 labeling of only <10% of tumor cells in an EcPV2-negative, lingual SCC (BOM), (**a**) bar = 80 µm, (**b**) bar = 40 µm. (**c**,**d**): Mild to moderate, diffuse, membranous CD271 labeling in an EcPV2-positive, oral SCC (VLU). This horse has a history of penile SCC harboring the same genetic EcPV2 variant as the oral tumor, (**c**) bar = 80 µm, (**d**) bar = 40 µm. (**e**,**f**): Central CD271 labeling is likewise observed in sections of this oral SCC (VLU), (**e**) bar = 80 µm, (**f**) bar = 40 µm. (**g**,**h**): CD271 positive (**g**) and negative control (**h**) (equine SCC ± primary Ab), bar = 100 µm. Framed areas are presented at higher magnification. (**i**): EMT in a lingual SCC, as revealed by CD271-staining. Transition of tumor cells of typically polygonal epithelial phenotype into spindle-type cells (encircled area) within the infiltration front of an EcPV2-negative, metastasizing lingual SCC (BOM). Bar = 100 µm.

The nerve growth factor receptor known as CD271 (or p75^NTR^) is a member of the tumor necrosis factor receptor (TNFR) superfamily. In recent years, CD271 has emerged as a promising marker for specific identification of CSC subpopulations in several types of human solid cancers including HN cancers [32,51]. In most tumor sections (17/22), CD271 labeling was mild to moderate, with a diffuse or patchy distribution. Focally intense CD271 labeling was noted in tumor sections derived from two EcPV2-positive (MAL, SIR) horses and one EcPV2-negative (SHM) horse. Centrally accentuated CD271 labeling patterns were noted in two EcPV2-positive cases (PRI, VLU) (Table 3; Figure 7a–f). An equine SCC previously established as a positive control exhibited Ab-binding to the CD271 antigen (Figure 7g), whilst mock labeled sections scored negatively (Figure 7h).

### 2.4. Immunofluorescent Double-Staining of HNSCCs for Keratins and Vimentin Reveals pEMT 

**Figure 8:**
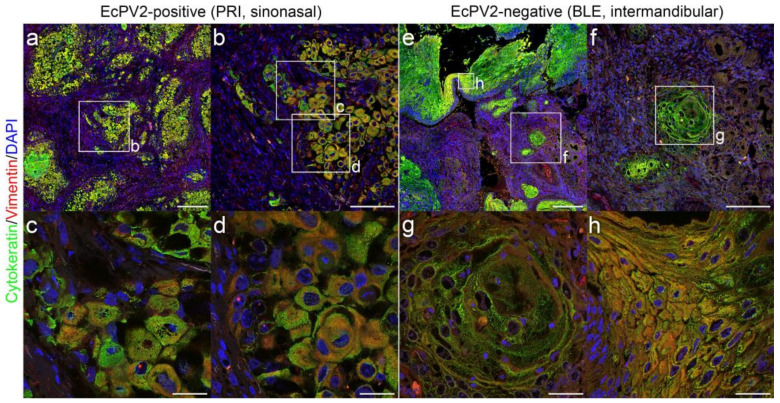
IF double-staining reveals KRT^+^ vimentin^+^ tumor cells consistent with pEMT. Overview: (**a**,**e**), 20× magnification: (**b**,**f**), and 63× magnification: (**c**,**d**,**g**,**h**) images of representative HNSCC sections (PRI, EcPV2-positive, sinonasal SCC: top panels, respectively; BLE, EcPV2-negative intermandibular SCC: bottom panels, respectively). Framed areas (**c**,**d**,**g**,**h**) depict representative sites of EMT and pEMT shown enlarged in the lower row. Immunofluorescent staining revealed cytoplasmic labeling of KRT (green) and vimentin (red). Note the yellow–orange double-positive cells representing mesenchymal transdifferentiated epithelial cells (pEMT). Nuclei were visualized by DAPI (blue). Scale bar = 250 µm (**a**,**e**), 100 µm (**b**,**f**), 25 µm (**c**,**d**,**g**,**h**).

Single immunohistochemical analysis of EcPV2-positive and negative HNSCCs yielded KRT labeling in all lesions to different extents, with varying labeling intensities and patterns (Figure 2; Table 2). Vimentin labeling revealed variable amounts of positive cells that were patchily distribution in most cases (Table 2; Figure 4). To further analyze EMT by studying the localization of KRT and vimentin expression in more detail, we subjected EcPV2-positive and EcPV2-negative tumor sections to double IF staining for these molecules. KRT and vimentin co-expression was noted in all HNSCC sections examined. Signals localized at the infiltrative fronts of the tumors or were irregularly distributed. Representative KRT/vimentin double-staining results from EcPV2-positive (Figure 8a–d) and EcPV2-negative (Figure 8e–h) tumors are depicted in Figure 8.

### 2.5. Immunofluorescent Double-Staining of HNSCC Sections Reveals CD44^+^ CD271^+^ Tumor Cells 

**Figure 9:**
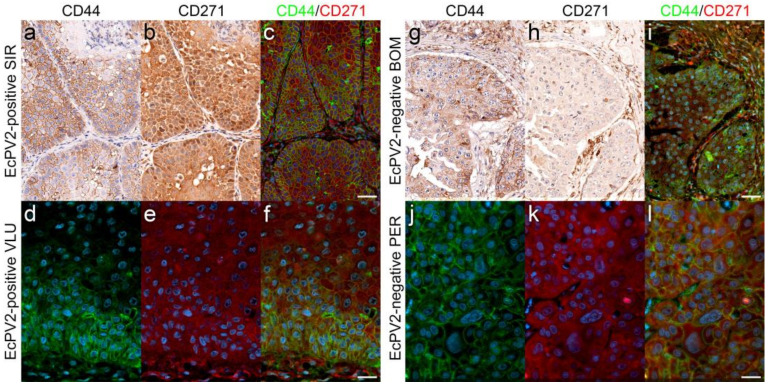
IF double-staining revealed CD44^+^CD271^+^ tumor cell subsets. Top: Sections of EcPV2-positive (SIR, VLU; left panels) and EcPV2-negative (BOM, PER; right panels) HNSCCs were comparatively assessed for single expression of CD44 (**a**,**g**) and CD271 (**b**,**h**) by IHC, and double expression of these stem-cell markers (**c**,**i**) by IF. IHC-staining revealed confinement of CD44 expression to the cell surface (**a**,**g**), whilst CD271 expression was cytoplasmic (**b**,**h**), with varying signal intensities and distributions. High CD44 and CD271 expression within tumor margins was observed for sections of the EcPV2-positive HNSCC (SIR) (**a**–**c**) in contrast to diffuse, mild to moderate expression in the EcPV2-negative tumor (**g**–**i**). Bottom: IF single staining for CD44 (**d**,**j**) and CD271 (**e**,**k**) and double-staining for both stem-cell markers (**f**,**l**) revealed a subset of CD44^+^CD271^+^ tumor cell subpopulations. Whereas double staining was most pronounced within the infiltration front in an EcPV2-positive HNSCC (VLU; (**d**–**f**)), the double-signal distribution seemed to be more disorganized in the EcPV2-negative lesion (PER; (**j**–**l**)). Diaminobenzidine chromogen was used for IHC staining, and hematoxylin counterstaining was performed to visualize cell nuclei. In the IF-based experiment, CD44-Alexa 488 produced a green, and CD271-Alexa 568 a red signal. Nuclei were visualized by DAPI (blue). Scale bar = 50 µm (top panels); scale bar = 20 µm (bottom panels).

In human HNSCCs, CSCs have been previously described as a CD44^+^CD271^+^ tumor cell subpopulation within the CD44^+^ compartment [52]. Given that single CD44- and CD271 staining revealed the presence of variable amounts of positive cells in all tumor sections analyzed (Table 3), we finally screened HNSCC sections for CD44^+^CD271^+^ tumor cells in an IF double-staining approach. Signal co-localization was observed with variable intensity in virtually all EcPV2-positive and -negative tumors. In some cases, CD44^+^CD271^+^ cells were predominantly detected within tumor margins representing infiltrative tumor fronts. In other cases, the CD44^+^CD271^+^ staining patterns were less organized, irrespective of tumor differentiation. Representative results are depicted in Figure 9.

## 3. Discussion

In humans, HNSCC constitutes a potentially lethal disease. However, early diagnosis and treatment of HNSCC precursor lesions such as plaques or papillomas can help prevent the development of late-stage lesions and metastasis [53]. In horses and other equids, benign SCC precursor lesions usually remain unnoticed when affecting the HN region. Owners commonly consult a veterinarian when noticing ingestion problems, head deformity, nasal discharge, weight loss, or ataxia. This leads to disease being diagnosed when precursor lesions have already progressed to large tumor masses invading adjacent tissue and bone, and impairing physiological functions [54]. At this stage, euthanasia usually represents the only ethically justifiable escape strategy [54,55,56]. In cases where disease is accidently detected at an earlier stage, e.g., during routine dental examination, the therapeutic repertoire is still very limited, with surgical tumor excision constituting the most common approach [55]. The considerable lack of therapeutic alternatives is due to the location of the tumor within the complex, highly innervated, and interrelated compartments of the HN region [57], and the poor understanding of the mechanisms underlying HNSCC development and progression in the horse.

To help pave the way towards a better understanding of the pathobiology of equine HNSCC, we first screened 49 tumors with confirmed diagnosis of HNSCC for the presence of equine papillomavirus infection. We opted to target EcPV2, EcPV3, and EcPV5, as these EcPV types have already been reported in association with muco-cutaneous lesions [9]. Notably EcPV2 DNA and transcripts were previously detected in HNSCCs by several groups [12,13,40,58,59]. In agreement with these findings, EcPV2 PCR yielded amplicons of expected size in 22% (11/49) of cases, whilst EcPV3 and EcPV5 PCRs scored consistently negative. Despite the still limited number of equine HNSCCs tested for EcPV2 infection so far, the herein-reported EcPV2 detection rate of 22% suggests that similar proportions of equine and human HNSCCs are PV-related [1]. Together with the previously reported finding of EcPV2 infection in <10% of apparently healthy equids [9,13,60,61], detection of EcPV2 DNA in a subset of HNSCCs points to a causal association of viral infection with these tumors. In-depth in vitro and ex vivo studies are beginning to help to clarify this issue.

Recently, Armando et al. reported on the detection of EcPV2 DNA from a laryngeal SCC affecting an elderly Maremmano mare. The group provided evidence of viral E6 oncogene transcription, indicating that EcPV2 was actively involved in neoplastic cell transformation, and by this, in disease progression [40]. In addition, this group was the first to address intralesional EMT in an equine case of HNSCC. The reported cadherin switch, and expression of EMT-associated transcription factors TWIST-1, ZEB-1, and HIF-1α were highly indicative for EMT events in the tumor, possibly explaining the metastatic behavior [40]. In the herein-presented study, we assessed 11 EcPV2-positive and 11 EcPV2-negative tumors with confirmed diagnosis of (metastasizing) HNSCCs for expression of selected EMT and CSC markers using IHC and IF.

Intratumoral presence of the epithelial tumor cell phenotype was assessed by immunohistological staining for keratins (KRTs) and β-catenin. All tumor sections scored consistently positive for KRT expression, with up to 100% of tumor cells displaying a cytoplasmic signal. In many lesions, KRT-staining patterns reflected the high degree of tissue disorganization, as recently described for EcPV2-positive equine penile SCCs and three-dimensional rafts established therefrom [62]. Over 50 to 100% of tumors cells also scored positive for β-catenin expression that was predominantly confined to the cell membrane irrespective of the EcPV2 infection status. This finding contrasts with other reports describing translocation of β-catenin expression to the nucleus in human and equine HNSCC cells [40,46,47,63]. Nuclear translocation results in β-catenin acquiring tumor-promoting properties by activating the expression of various oncogenes such as fibronectin, cyclin D1, or c-myc, leading to deregulation of cell-cycle progression [47,64]. Interestingly, nuclear β-catenin translocation is particularly observed in hrHPV-induced carcinomas. There are indications that membranous β-catenin expression in favor of nuclear expression of the protein is mediated by overexpressed E6 and E7 [47]. On these grounds, it may be speculated that E6 and E7 expression levels in EcPV2-positive equine HNSCCs were too low to allow for interference with β-catenin expression.

EMT is characterized by the so-called cadherin switch, i.e., downregulation of E-cadherin and upregulation of N-cadherin. Loss of E-cadherin entails the loss of epithelial KRTs, and expression of the mesenchymal protein vimentin, providing the tumor cells with migratory properties [16,19]. In the equine HNSCCs analyzed, vimentin was expressed by tumor cells to a various extent in 100% of lesions, irrespective of the precise tumor location and the EcPV2 infection status. This finding agrees with the histopathological classification of all tumors as late-stage lesions, with metastasis confirmed in 10/22 lesions subjected to expression analyses. Overall, vimentin-staining revealed a patchy distribution within tumor islets. Pronounced confinement of vimentin expression to neoplastic cells of the infiltration front was observed in a single EcPV2-positive case. This contrasts with reported occasional expression of vimentin only in the infiltratively growing portion of EcPV2 associated penile SCCs [39]. To further characterize EcPV2-positive and –negative HNSCCs with respect to the localization of KRT and vimentin expression, we analyzed tumor sections by IF KRT/vimentin double-staining and generated high-resolution images. The latter provided robust evidence of KRT^+^vimentin^+^ tumor cells in all lesions that very likely represent E/M hybrid cells that had undergone pEMT. This finding agrees with the concept that epithelial tumor cells do not complete EMT in vivo, but rather remain in a pEMT state in human patients [24]. This state is characterized by simultaneous expression of epithelial- and mesenchymal-type proteins providing E/M hybrid cells with the abilities to attach and migrate, and hence, to migrate collectively [24,25]. The observation that equine HNSCC cells had undergone pEMT rather than complete EMT also provides a sound explanation for the predominantly membranous expression of β-catenin by tumor cells.

COX-2 is a cyclooxygenase isoform that acts as inflammatory enzyme in chronic inflammation. Importantly, there is substantial evidence that COX-2 overexpression orchestrates EMT via creation of an inflammatory tumor microenvironment [65]. In equine HNSCC, COX-2 expression was consistently detected, with <10 up to >50% of tumor cells staining positive. Although statistically not significant at *p* < 0.1, there was a tendency of EcPV2-positive HNSCCs harboring more COX-2 positive tumor cells than their EcPV2-negative counterparts. This observation is in accordance with the finding of HPV type 16 E6 and E7 oncoproteins promoting COX-2 overexpression [66].

EMT is also associated with a second program promoting the transition of epithelial-type tumor cells to CSCs. The latter are characterized by MDR, and also known as tumor-initiating cells (TICs) due to their intrinsic ability to form tumors in vivo, and promote tumor growth, recurrence, and metastasis [67]. In human HNSCCs, the presence of CSCs is well-documented, and their frequency positively correlates with severity of disease [51,52,68,69]. In horses and other equids, no information is available on CSCs in any tumor disease. This substantial lack of knowledge prompted us to put a special focus on the detection of CSCs in equine HNSCC.

Since the discovery of CSCs, CD44 has evolved as useful marker for detection and isolation of this particular tumor cell subset [51]. CD44 labeling of equine HNSCC sections revealed the presence of CD44^+^ tumor cells in 100% of tumors analyzed, with positive tumor cells ranging between >10 and >50%. A lower percentage was only determined for a periocular tumor. Of note, CD44 is also expressed by a wide range of immune cells that reside in HNSCCs as infiltrates and are also part of the tumor stroma [51]. This fact was kept in mind when assessing the numbers of CD44^+^ tumor cells.

In the past two decades, another surface molecule, i.e., CD271, has been identified as potent CSC marker in human melanoma [70] and HNSCC [71,72,73]. Labeling of equine HNSCCs revealed CD271 expression in up to 100% of tumor cells. This finding is not completely surprising, since CD271 is also expressed by undifferentiated cells in normal epithelium [51]. It can be speculated, that both undifferentiated tumor cells and CSCs may express CD271 in equine HNSCC. Since the specificity of all antibodies was evaluated prior to this study, high CD271 labeling due to unspecific binding appears rather unlikely.

Combined use of CD44 and CD271 markers in HNSCC research recently led to the identification of CSCs as a CD271^+^ subpopulation within the CD44^+^ tumor cell compartment [51,52,68,69]. Based on this intriguing discovery, we subjected equine HNSCC section to immunofluorescent CD44/CD271 double staining, revealing the intralesional presence of CD44^+^CD271^+^ tumor cells in all tumors analyzed. This finding provides the first evidence of CSCs in high-grade equine SCCs affecting different parts of the HN region.

When comparing EMT and CSC marker expression intensities and rates in EcPV2-positive versus -negative equine HNSCCs, no significant differences attributable to presence or absence of EcPV2 infection were noted. This was somewhat disappointing as we expected to disclose some distinctive features, e.g., in relation to nuclear translocation of β-catenin in EcPV2-positive tumors, as described for hrHPV-induced versus -unrelated HNSCCs [47] or CSC percentages. Sole inclusion of late-stage equine HNSCCs in the study may represent a limiting factor, since differences regarding (p)EMT/CSC-induced malignant progression of EcPV2-related versus -unrelated lesions may be particularly encountered in premalignant HNSCC precursor lesions. Such differences, if existent, would determine the fate of these lesions. In addition, comparison of tumors affecting the same location within the HN region would certainly help obtain a more concise picture. More in-depth research is needed to elucidate the pathobiological role of EcPV2 in equine HNSCCs and related precursor lesions, with particular focus on the impact of E6 and E7 oncoprotein expression on (p)EMT-mediated tumor cell plasticity.

To conclude, this study provides evidence of (p)EMT constituting a common event in partly metastasizing equine EcPV2-positive and -unrelated HNSCCs, with epithelial tumor cells adopting an E/M hybrid or CSC phenotype. The observed phenotype switching emphasizes the high tumor cell plasticity in equine HNSCC and provides a sound explanation for the malignancy and potential metastatic behavior of the disease.

## 4. Method

### 4.1. Sample Material

A total of 49 histopathologically confirmed equine HNSCC were included in the study. Archival, formalin-fixed paraffin-embedded (FFPE) tumor material was available in 49/49, native tumor material and whole DNA extracted therefrom in 16/49 cases. All tumor samples were collected at the Veterinary University of Vienna, Austria, during therapeutic surgical excisions or during requested necropsies with the owners’ written consent. Patient, disease, and sample specifications are provided in Table 1.

### 4.2. DNA Extraction

DNA aliquots from 15/16 native tumor samples were already available [13]. The one additional native sample (VLU) was subjected to DNA extraction using a DNeasy Blood & Tissue Kit (Qiagen, Hilden, Germany) according to instructions of manufacturer. Obtained DNA was stored at −20°C until use.

To prepare FFPE samples for DNA isolation, 10-µm tissue sections were deparaffinized according to an established protocol [74]. In brief, sections were vortexed with 1 mL xylene (Merck, Darmstadt, Germany) and incubated at room temperature for 5 min. Following a centrifugation step at 13,000 × g for 5 min, supernatants were discarded, and the procedure was repeated. Then, resulting pellets were vortexed in 1 mL 96% ethanol each (Merck, Darmstadt, Germany) and centrifuged at 13,000 × g for 5 min. Supernatants were discarded and the washing procedure was repeated. In a final step, ethanol was removed, and pellets were dried under vacuum using a desiccator for up to 1.5 h. Subsequently, pellets were subjected to DNA extraction as described above.

### 4.3. EcPV PCR

Following assessment of DNA concentrations per photometry, equine β-actin PCR was performed to test all DNA isolates for PCR compatible quality. Reactions were carried out as described previously [75], with the only difference that the published forward primer was combined with reverse primer 3′ β-actin-683 (5′- gccatctcttgctcgaagtccagg-3′) for generation of a shorter amplification product (208 bp) to assure successful amplification also from FFPE-derived template DNA. Given that β-actin PCR scored positive for all DNA isolates, the latter were subsequently assessed for the presence of EcPV types 2, 3, and 5 using type-specific PCR. The primer pairs used for amplification of a 173 to 270 bp region within the respective E6 or E7 open reading frames were the following: 5′ and 3′EcPV2 E7 (5′-ggatcctgcagcaactgc-3′; 5′-atcactatcacagtcgctacacagc-3′; product size: 173 bp), 5′and 3′EcPV3 E6 (5′-ctgttgaagctcgctactgagtcac-3′; 5′-gtctccactgcttctccctaaactc; product size: 270 bp), and 5′and 3′EcPV5 E6 (5′-cgctacagcggggacgac-3′; 5′-ggaggtgagcagtgacgaagag-3′; product size: 257 bp). Reactions were conducted with Thermo Scientific™ Phusion Hot Start II DNA-Polymerase (Fisher Scientific GmbH, Schwerte, Germany), or PCRBIO HS VeriFi™ Polymerase (PCR Biosystems Ltd., London, UK) according to instructions of the manufacturer. The amplification program consisted of an initial denaturation step at 95 °C for 5 min, followed by 40 cycles (95 °C for 15 sec, 67 °C for 30 sec, 72 °C for 45 sec), and a final elongation step at 72 °C for 5 min. Amplification products (16 µL) were subjected to gel-electrophoresis using 2%TAE agarose gels, and visualized by ethidium bromide staining.

Every PCR reaction included a positive (EcPV type 2-, 3- or 5-positive equine DNA), a negative (PV-free equine DNA), and a no-template control (ntc; sterile water).

### 4.4. Histopathological Examination

We matched 11 EcPV2-positive HNSCC with 11 EcPV2-negative HNSCC according to the grade of differentiation, the anatomical tumor location, and, as far as possible, the patient’s age. Hematoxylin- and eosin-stained (HE) sections of FFPE tissue material of selected cases were histopathologically re-examined for verification of sample matching. To characterize the degree of histological differentiation, archived FFPE tissue from necropsies and biopsies were evaluated with respect to tumor cell morphology and grade of cornification.

### 4.5. Immunohistochemical Staining (IHC)

In a first step, fresh 2.5 µm-sections of 11 EcPV2-positive and 11 EcPV2-negative tumor FFPE samples were assessed by a single labeling approach for expression of keratins (KRT), β-catenin, vimentin, COX-2, CD271 (p75^NTR^), and CD44. To this end, sections were deparaffinized with xylene, and successively dehydrated in 100%, 96%, and 70% ethanol. Then, sections were treated with 0.3% H_2_O_2_/methanol to block peroxidase activity. Heat-induced epitope retrieval (HIER) was performed in 0.1 M citrate buffer (pH 6) or TRIS-EDTA (pH 9; Table 2) for 30 min in a steamer at 94–100 °C. To minimize unspecific binding, slides were blocked with 1.5% normal goat serum. Incubation with primary antibody (Ab; for Ab specifications see Table 4) was conducted at 4 °C overnight. After washing with phosphate-buffered saline (PBS), sections were incubated with secondary horseradish peroxidase (HRP)-conjugated Ab (Table 4) for 30 min at room temperature. Ab-bound protein was visualized with diaminobenzidine (DAB) chromogen. Hematoxylin (HE) was used for nuclear counterstaining. Evaluation of signals was semiquantitatively performed by blinded investigator AK using an Olympus BX45 light microscope. The following staining characteristics were assessed: (i) intracytoplasmic versus membranous immunostaining; (ii) DAB signaling intensity that was classified as absent (negative), mild, moderate, or strong.; (iii) the labeling pattern, i.e., diffuse labeling of all tumor cell layers, patchy labeling with irregular distribution, and special labeling patterns in the center of tumor islets or the infiltrative front; and (iv) estimated percentage of positive tumor cells (<10%, <50%, and >50%). For evaluation of larger tissue samples, five highly representative fields were selected in 100x magnification. Images were captured using an Olympus BX51 microscope equipped with an Olympus camera UC90 (Olympus, Vienna, Austria). High-resolution images were captured using a Zeiss LSM880 Airyscan confocal microscope (Carl Zeiss AG, Jena, Germany).

### 4.6. Immunofluorescent Staining (IF)

In a second step, sections were subjected to KRT/vimentin and CD44/CD271 double immunofluorescent (IF) staining. Sections were rehydrated and pretreated with TRIS-EDTA-buffer at pH9 for 30 min in the steamer for epitope retrieval as described above. Following blocking with goat serum, sections were incubated with mixtures of primary anti-KRT and anti-vimentin, or anti-CD44 and anti-CD271 Abs (Table 4). Alexa Fluor® (Thermo Fisher Scientific, Vienna, Austria) 488 (green signal) and 568 (red signal) conjugated Abs were used as secondary Abs (Table 4). Following incubation with 4′, 6-diamidino-2-phenylindole (DAPI) for nuclear counterstaining and rinsing with water, slides were mounted with Aqua-PolyMount (Polysciences, Szabo-Scandic, Vienna, Austria) and digitized using a Panoramic Scan II Slide scanner (3DHistech, Budapest, Hungary).

### 4.7. Statistical Analyses

The significance of differences between respective labeling intensities and % positive cells in EcPV2-positive versus -negative HNSCC sections were assessed by the Mann–Whitney U test (https://www.socscistatistics.com/tests/mannwhitney/; accessed on 10 February 2022). Statistical significance was set at *p* < 0.1.

## Figures and Tables

**Table 1:** Patient and sample specifications including EcPV2 PCR results.

Code	Breed	Age	Color	Sex	Sample Origin/Histopathological Diagnosis	Cornification	Native, FFPE	EcPV2 DNA
**EQUINE HNSCC PATIENTS AND TUMOR SAMPLES THEREFROM SELECTED FOR IHC ANALYSIS (n = 22)**								
DAN	WB	22	Chestnut	G	Nasal and oral SCC, retropharyngeal LN metastases	+	N, F	YES
MAL	Arabian TB	19	Grey	M	Nasal SCC with orbital infiltration	+	F	YES
VAL	Icelandic horse	18	Black	M	SCC of dorsal nasal concha, osteolysis	++	N, F	No
DIA	Trotter	17	Black	M	SCC of the left nasal cavity and paranasal sinus, osteolysis	+	N, F	No
MMO	Haflinger mix	13	Bay	G	Sinus SCC	No	F	No
PRI	Shetland pony	26	Black	G	Nasal SCC	++	N, F	YES
HJA	Icelandic horse	16	Bay	G	Maxillary sinus SCC, oral and retropharyngeal LN metastases	+	F	YES
KLU	Noriker horse	17	Piebald	G	Maxillary sinus SCC invading lymphatics and blood vessels	+	F	YES
FIL	WB	26	Chestnut	G	Maxillary sinus SCC	No	N, F	No
PER	Trotter	20	Bay	G	Maxillary sinus SCC, mandibular and retropharyngeal LN metastases	+	F	No
SHM	Cob	23	Piebald	G	Sinonasal SCC	++	F	No
MEC	Shetland pony	25	Black	M	Oral SCC right mandibula, maxilla	++	F	No
BLE	WB	11	Piebald	M	Intermandibular SCC (recurrent lesion)	+	F	No
SHA	Pinto	17	Skewbald	M	Lingual and intermandibular SCC	+	N, F	YES
BOM	WB	24	Bay	M	Lingual SCC, retropharyngeal and tracheal LNs metastases	No	F	No
JON	Pony	25	Chestnut	G	Gingival/palatal SCC	No	N, F	YES
LUK	WB	17	Grey	G	Palatal SCC invading maxillary sinus	++	F	No
SIR	WB	11	Fuchs	M	Mandibular SCC	++	F	YES
KRA	Icelandic horse	21	Fuchs	G	Oral SCC, regional LNs metastases	No	F	No
VLU	Icelandic horse	30	Chestnut	G	Oral SCC	++	N, F	YES
SAM	Haflinger-WB	13	Sorrel	G	Pharyngeal SCC, retropharyngeal LN metastases	+	N, F	YES
MCH	Connemara	15	Grey	M	Periocular SCC with metastases (parotis, larynx, jugular notch)	+	N, F	YES
**EQUINE HNSCC PATIENTS AND TUMOR SAMPLES THEREFROM (NO IHC ANALYSIS) (n = 27)**								
MIL	WB	22	Chestnut	M	Maxillary sinus SCC	+	N, F	No
GER	Trakehner	18	Black	G	Sinonasal SCC with mandibular LN metastases	No	N, F	No
STA	Haflinger	7	Sorrel	G	Nasal SCC, mandibular LN metastases	No	N, F	No
ATH	Haflinger	22	Sorrel	G	Oral vestibule SCC	+	N, F	No
BEL	Haflinger	22	Sorrel	M	Paranasal sinus SCC with pronounced osteolysis	No	N, F	No
JAN	Hungarian WB	17	Bay	M	Maxillary sinus SCC, retropharyngeal LN metastases	++	F	No
CAT	Trotter	7	Bay	M	Sinonasal SCC, osteolysis right maxilla	+	F	No
CHI	Trotter	23	Bay	M	Maxillary SCC invading oral cavity and brain	No	F	No
ANG	Haflinger	16	Chestnut	M	Maxillary SCC, suspected metastatic activity	No	F	No
SNI	Connemara	16	Grey	M	Mandibular SCC invading lymphatics, LN metastases	No	N, F	No
PEN	Shetland pony	26	Piebald	M	Mandibular SCC, osteolysis	++	F	No
ARR	TB	21	Bay	M	Mandibular SCC left, osteolysis	+	N, F	No
FEX	Pony	27	Sorrel	G	Mandibular SCC invading tongue, trachea, esophagus	No	F	No
GAJ	Trotter	13	Bay	G	Palatal/gingival SCC, bone arrosion	+	N, F	No
WHI	WB	20	Skewbald	M	Ulcerative, lingual SCC, invading left stylohyoid	++	F	No
IAS	Arabian TB	22	Grey	M	Lingual SCC, retropharyngeal LN metastases	++	F	No
NEP	Lusitano	21	Grey	G	Lingual base SCC	+	F	No
MOZ	WB	27	Grey	G	Lingual base SCC, retropharyngeal LNs metastases	+	F	No
KAR	Haflinger	23	Sorrel	M	Lingual SCC, retropharyngeal, mandibular, cranial LN metastases	+	F	No
GOL	Haflinger	23	Sorrel	G	Labial SCC (+conjunctival and cutaneous CIS)	++	F	No
NAV	Haflinger	17	Sorrel	G	Labial SCC (+right eye: CIS; left eye: SCC)	++	N, F	No
JOY	Haflinger	20	Sorrel	M	Labial SCC (recurrent lesion), mandibular LN metastases	+	F	No
LEN	WB	23	Chestnut	M	Maxillary SCC, mandibular LN metastases	+	F	No
RIG	Haflinger	9	Sorrel	G	Mandibular and labial SCC, mandibular LN metastases	+	F	No
GRA	Arabohaflinger	20	Sorrel	G	Nasal CIS	++	F	No
ARM	Haflinger	12	Sorrel	M	Cutaneous perinasal CIS (+conjunctival SCC)	No	F	No
DAL	Trotter	12	Bay	G	Paranasal sinus and oral SCC	+	F	No

SCC: squamous cell carcinoma; CIS: carcinoma in situ; G: gelding; M: mare; N: native; FFPE: Formalin-fixed paraffin-embedded; LN(s): lymph node(s). ++: cornified; +: partly cornified, No: no cornification.

**Table 2:** Compiled β-catenin, vimentin, and keratin single-labeling results.

HNSCC (n = 22)		Keratin				β-Catenin			Vimentin			
Code	Tumor Analyzed	I	D	% +Cells	P	I	D	% +Cells	I	D	% +Cells	P
*DAN*	*nasal*	*1–3*	*diffuse*	*≤* *100*	*central+*	*3*	*diffuse*	*≤* *100*	*3*	*patchy*	*<50*	
*MAL*	*nasal*	*2–3*	*diffuse*	*≤* *100*		*3*	*diffuse*	*≤* *100*	*3*	*patchy*	*<10*	
VAL	sinonasal	3	diffuse	≤100		1–2	diffuse	≤100	3	patchy	<10	
DIA	sinonasal	1–2	diffuse	≤100	central+	1	patchy	>50	3	patchy	<10	
MMO	sinonasal	2–3	diffuse	≤100		2	diffuse	≤100	1–3	patchy	<50	
*PRI*	*sinonasal*	*3*	*diffuse*	*≤* *100*		*3*	*diffuse*	*≤* *100*	*2–3*	*patchy*	*<50*	*marginal*
*HJA*	*sinonasal*	*1–3*	*diffuse*	*≤* *100*		*1–3*	*diffuse*	*≤* *100*	*3*	*patchy*	*<10*	
*KLU*	*maxillary sinus*	*1–2*	*diffuse*	*≤* *100*		*1–3*	*patchy*	*≤* *100*	*1–3*	*patchy*	*>50*	
FIL	maxillary sinus	3	diffuse	≤100		1	diffuse	≤100	3	patchy	>50	
PER	maxillary sinus	2–3	diffuse	≤100	central+	2–3	diffuse	≤100	2–3	patchy	<50	
SHM	sinus	2–3	diffuse	≤100	central+	3	patchy	≤100	3	patchy	<50	
MEC	mandibular	2–3	diffuse	≤100	central+	1–2	patchy	>50	2–3	patchy	<50	
BLE	intermandibular	2–3	diffuse	≤100	central+	3	diffuse	≤100	3	patchy	<10	
*SHA*	*linguomandibular*	*3*	*diffuse*	*≤* *100*		*3*	*diffuse*	*≤* *100*	*3*	*patchy*	*<10*	
BOM	lingual	3	diffuse	≤100		1	diffuse	≤100	3	patchy	<10	
*JON*	*gingivopalatal*	*1–3*	*diffuse*	*≤* *100*	*c* *entral+*	*1*	*diffuse*	*≤* *100*	*3*	*patchy*	*<10*	
LUK	palatal	3	diffuse	≤100		2	diffuse	≤100	3	patchy	<50	
*SIR*	*labiopalatal*	*1–2*	*diffuse*	*≤* *100*	*central+*	*2*	*diffuse*	*≤* *100*	*3*	*patchy*	*<10*	
KRA	oral	2–3	diffuse	≤100		1–2	patchy	>50	3	patchy	<10	
*VLU*	*oral*	*1–2*	*diffuse*	*≤* *100*	*central+*	*1*	*patchy*	*>50*	*1*	*patchy*	*<10*	
*SAM*	*laryngeal*	*3*	*diffuse*	*≤* *100*		*3*	*diffuse*	*≤* *100*	*3*	*patchy*	*<10*	
*MCH*	*periocular*	*3*	*diffuse*	*≤* *100*		*1*	*diffuse*	*≤* *100*	*3*	*patchy*	*<10*	

I: intensity, D: distribution, and P: pattern of labeling; *Italics*: EcPV2-positive tumors.

**Table 3:** Compiled COX-2, CD44, and CD271 single-labeling results.

HNSCC (n = 22)		COX-2			CD44			CD271			
Code	Tumor Analyzed	I	% +Cells	P	I	D	% +Cells	I	D	% +Cells	P
*DAN*	*nasal*	*0–3*	*>50*		*0–2*	*patchy*	*>50*	*1–2*	*diffuse*	*≤* *100*	
*MAL*	*nasal*	*0–3*	*<50*		*0–1*	*patchy*	*<50*	*1–3*	*diffuse*	*≤* *100*	
VAL	sinonasal	0–3	<10	marginal	0–2	patchy	<50	1	diffuse	≤100	
DIA	sinonasal	0–1	<10		0–2	patchy	<50	0–1	diffuse	>50	
MMO	sinonasal	0–3	>50		0–3	patchy	>50	1	diffuse	≤100	
*PRI*	*sinonasal*	*2–3*	*>50*		*1–2*	*diffuse*	*na*	*0–2*	*diffuse*	*>50*	*central+*
*HJA*	*sinonasal*	*0–3*	*>50*		*0–1*	*patchy*	*<50*	*1–2*	*diffuse*	*≤* *100*	
*KLU*	*maxillary sinus*	*0–3*	*<50*	*margi* *nal*	*0–3*	*patchy*	*>50*	*1–2*	*patchy*	*≤* *100*	
FIL	maxillary sinus	0–3	<50		0–2	patchy	<50	1–2	diffuse	≤100	
PER	maxillary sinus	0–3	<50	marginal	0–2	patchy	>50	1–2	diffuse	≤100	
SHM	sinus	0–3	>50		1–3	patchy	>50	1–3	diffuse	≤100	
MEC	mandibular	0–3	<50		0–3	patchy	>50	0–1	patchy	>50	
BLE	intermandibular	0–3	<50		1–2	patchy	<50	1–2	diffuse	≤100	
*SHA*	*linguomandibular*	*0–2*	*<50*		*0–2*	*patchy*	*<50*	*1–2*	*diffuse*	*≤* *100*	
BOM	lingual	0–1	<10		0–2	patchy	>50	0–1	patchy	>50	
*JON*	*gingivopalatal*	*0–3*	*<50*		*0–3*	*patchy*	*>50*	*1–2*	*diffuse*	*≤* *100*	
LUK	palatal	0–3	>50		0–2	patchy	>50	1–2	diffuse	≤100	
*SIR*	*labiopalatal*	*0–3*	*<50*		*0–2*	*patchy*	*>50*	*2–3*	*diffuse*	*≤* *100*	
KRA	oral	0–3	<50		0–1	patchy	<50	1	diffuse	≤100	
*VLU*	*oral*	*0–3*	*<10*	*marginal*	*0–2*	*patchy*	*>50*	*1–2*	*diffuse*	*≤* *100*	*central+*
*SAM*	*laryngeal*	*0–3*	*>50*		*1–3*	*patchy*	*<50*	*1–2*	*patchy*	*>50*	
*MCH*	*periocular*	*0–3*	*<50*		*0–2*	*patchy*	*<10*	*1*	*diffuse*	*100*	

I: intensity, D: distribution, and P: pattern of labeling; *Italics*: EcPV2-positive tumors.

**Table 4:** Antibody and pretreatment specifications.

Host	Type	Clone	Target Protein	Provider	Dilution	HIER
**IHC (single staining)**						
**Primary Abs**						
Mouse	Monoclonal	AE1	LMW keratins	Cell Marque, Sigma-Aldrich, Vienna, Austria	1:650	pH 9
Mouse	Monoclonal	AE3	HMW keratins	Cell Marque, Sigma-Aldrich, Vienna, Austria	1:650	pH 9
Mouse	Monoclonal	9G2	Beta-catenin	Acris Antibodies, Herford, Germany	1:500	pH 9
Mouse	Monoclonal	V9	Vimentin	Dako, Hamburg, Germany	1:500	pH 6
Rabbit	Recombinant	EPR3208	CD146	Abcam, Cambridge, UK	1:500	pH 6
Rabbit	Monoclonal	SP21	COX2	Thermo Fisher Scientific, Vienna, Austria	1:400	pH 6
Rat	Monoclonal	IM7	CD44	Santa Cruz Biotechnology, Dallas, Texas, USA	1:200	pH 6
Rabbit	Monoclonal	D4B3	CD271 (p75NTR)	Cell Signaling Technology, Frankfurt, Germany	1:1000	pH 9
**Secondary Abs**						
BrightVision Poly-HRP anti-mouse Ab				ImmunoLogic, Duiven, The Netherlands		
BrightVision Poly-HRP anti-rabbit Ab				ImmunoLogic, Duiven, The Netherlands		
Goat anti-rat HRP conjugated				Abcam, Cambridge, UK		
**IF (double staining)**						
**Primary Abs**						
Mouse	Monoclonal	AE1	LMW keratins	Cell Marque, Sigma-Aldrich, Vienna, Austria	1:400	pH 9
Mouse	Monoclonal	AE3	HMW keratins	Cell Marque, Sigma-Aldrich, Vienna, Austria	1:400	pH 9
Rabbit	Polyclonal		Vimentin	Sigma Prestige, Merck, Vienna, Austria	1:500	pH 9
Rat	Monoclonal	IM7	CD44	Santa Cruz Biotechnology, Dallas, Texas, USA	1:500	pH 9
Rabbit	Monoclonal	D4B3	CD271 (p75^NTR^)	Cell Signaling Technology, Frankfurt, Germany	1:250	pH 9
**Secondary Abs**						
Donkey anti-mouse Ab A488 1:800				Jackson ImmunoResearch Europe, LTD, Ely, UK		
Donkey anti-rabbit Ab A568 1:400				Invitrogen, Thermo Fisher Scientific, Vienna, Austria		
Goat anti-rat Ab A488 1:800				Invitrogen, Thermo Fisher Scientific, Vienna, Austria		
Goat anti-rabbit Ab A568 1:1500				Invitrogen, Thermo Fisher Scientific, Vienna, Austria		

## Data Availability

All data are presented in this article.
